# Multiplex target capture with double-stranded DNA probes

**DOI:** 10.1186/gm454

**Published:** 2013-05-29

**Authors:** Peidong Shen, Wenyi Wang, Aung-Kyaw Chi, Yu Fan, Ronald W Davis, Curt Scharfe

**Affiliations:** 1 Stanford Genome Technology Center, Stanford University, Palo Alto, CA 94304, USA; 2 Department of Bioinformatics and Computational Biology, UT MD Anderson Cancer Center, Houston, TX 77030, USA

## Abstract

Target enrichment technologies utilize single-stranded oligonucleotide probes to capture candidate genomic regions from a DNA sample before sequencing. We describe target capture using double-stranded probes, which consist of single-stranded, complementary long padlock probes (cLPPs), each selectively capturing one strand of a genomic target through circularization. Using two probes per target increases sensitivity for variant detection and cLPPs are easily produced by PCR at low cost. Additionally, we introduce an approach for generating capture libraries with uniformly randomized template orientations. This facilitates bidirectional sequencing of both the sense and antisense template strands during one paired-end read, which maximizes target coverage.

## Background

Targeted next-generation sequencing (NGS) focuses on clinically actionable genes at both higher quality and lower cost than whole-genome sequencing [1,2], though it requires substantial improvements in performance, cost and multiplex sample processing to be applied in diagnostic molecular testing [3,4]. There are now several target enrichment strategies with the common goal to capture candidate genomic regions at high accuracy and completeness, while lowering the costs at the same time [2,5]. The most widely used methods utilize multiplex PCR amplification [6-8], hybrid-capture [9-12], selective target circularization [13-21], and oligonucleotide-selective sequencing [22]. Hybrid-capture methods are quickly scalable and have achieved high levels of uniformity in whole-exome sequencing (WES) [23], while circularization-based methods can provide increased sensitivity and specificity for variant detection in candidate gene resequencing compared to WES [14].

A common feature of these methods is their reliance on single-stranded oligonucleotides that serve as capturing probes during target enrichment. These probes can be synthesized chemically at high quality by column-based synthesis, or at relatively lower quality and cost using programmable microarrays [24]. The cost-effective synthesis of long (≥150mer) oligonucleotides at high yield and quality has remained most difficult [25]. This has been a limitation particularly for the construction of padlock probes and the molecular inversion probe (MIP) technology [24,26-28], which could benefit from probes that are much longer than what can be chemically synthesized. We previously developed a method for the construction of long padlock probes (LPPs), which are single-stranded DNA probes of approximately 320 bases in length that provide high target specificity through dual recognition of target sequences [17]. LPPs enabled capturing sequences from <100 to 500 bp in length, which was sufficient to capture most (>98%) exons in 524 genes using only a single probe [18]. To construct the single-stranded LPPs (ssLPPs) required removing one strand from a double-stranded precursor molecule using exonuclease digestion. This protocol consisted of a number of laborious steps of enzymatic digestions, de- and re-phosphorylation (Figure S1 in Additional file 1). In some cases, difficulty controlling probe quality and quantity during production led to a loss of probes. Here we report multiplex target capture with complementary LPPs (cLPPs), which are double-stranded probes that act as two independent single-stranded probes during target capture. cLPPs are easily produced through PCR and outperformed ssLPPs and can be built in less than half the time of previous probe construction.

We also address a second challenge for LPPs, which is the non-randomness in sequence capture libraries. In comparison, shotgun DNA sequencing (for example, whole-genome sequencing) utilizes libraries of randomly fragmented DNA [29], which is sequenced in both the forward and reverse direction during one paired-end (PE) read. This allows bidirectional sequencing of both DNA strands of target regions assuming even distribution and coverage of library fragments. Here we apply this concept to multiplex targeted sequencing to enable bidirectional targeted sequencing of both DNA template strands during one PE read. We show that our approach maximizes coverage uniformity across increasingly larger capture products and is fully compatible with existing library preparation protocols used by a variety of multiplex targeted sequencing methods.

## Materials and methods

### Selection of candidate genes and DNA samples

We selected 524 nuclear genes based on evidence for the localization of their gene products to human mitochondria and association to Mendelian disorders [18]. Genomic DNA for NA18507, NA12878 and NA03330 was obtained from the Coriell Institute for Medical Research (Camden, NJ, USA). Genomic DNA for a patient with ornithine transcarbamylase (OTC) deficiency and his healthy mother were obtained with informed consent and approved by the institutional review board at Stanford University. Research was carried out in compliance with the Helsinki Declaration.

### Construction of cLPPs and ssLPPs

The protocol of ssLPP construction by PCR amplification from lambda DNA was described previously [17,18]. In brief, oligonucleotide primers containing MlyI (forward primer) and/or BsaI (reverse primer) sites at their 5' ends, genomic target sequences (18 to 28 bp) in the middle, and bacteriophage lambda sequences at 3' ends, are used to amplify the probes' common spacer backbone of approximately 280 bp from bacteriophage lambda DNA. We generated 5,619 individual PCRs of approximately 350 bp in order to target all protein-coding sequences and flanking intronic regions of 524 genes (target size range <100 to 541 bp). All 5,619 PCR products were pooled in a single tube and purified through QIAquick columns (Qiagen, Valencia, CA, US). The purified PCR products are simultaneously digested for 1 hour at 37°C using MlyI and BsaI-HF (NEB, Ipswich, MA, USA), only leaving the target sequences at each probe end, respectively. Following QIAquick purification, the pool of cLPPs is ready for multiplex target capture. To generate ssLPPs, the pool of cLPPs required further enzymatic digestions as described before [17,18] and in Figure S1 in Additional file 1. In comparison, our new protocol for cLPP construction is greatly streamlined with improved probe yield and quality.

### Multiplex target capture with LPPs

**Figure 1:**
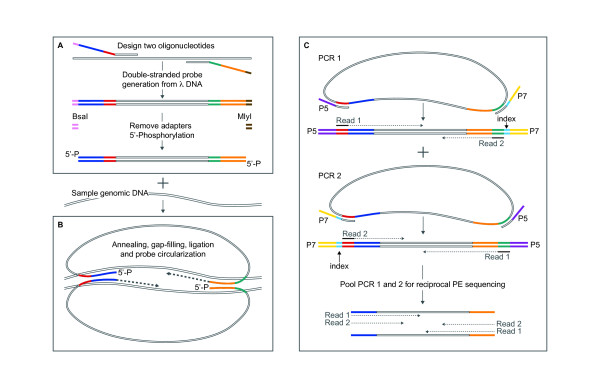
**Probe construction, target capture and reciprocal paired-end sequencing**. **(a) **Each cLPP contains a common linker flanked by post-capture amplification sites (red and green) and two target-specific capturing arms (blue and orange). Probe ends are trimmed (BsaI and MlyI) and 5'-phosphorylated to produce functional cLPPs. **(b) **Multiplex probe-target hybridization followed by gap-filling and ligation triggers probe circularization and target capture. **(c) **Capture libraries are multiplex-amplified using hybrid primers that anneal to the probes' amplification sites and add Illumina sequencing adaptors (P5 or P7) and sample-specific barcodes. This is done in two separate PCRs during which the adaptors swap positions at the ends of templates. Both PCRs are pooled for reciprocal PE sequencing of both DNA strands.

The amount of each probe can be adjusted according to the GC content of the target region, which we increased for high GC targets. Seventy femtomoles of cLPPs or ssLPPs (5 to 50 attomoles/probe) were mixed with 500 ng of genomic DNA (or WGA DNA) in 1× Ampligase buffer (Epicentre, Charlotte, NC, USA) and 0.9 M betaine (Sigma Aldrich, St Louis, MO, USA) in a 10 μl volume. The mixture was heated to 98°C for 3 minutes, followed by a gradual decrease in temperature of 1°C per minute to 56°C and held for 2 hours. For probe extension and ligation, a 10 μl mixture of 0.3 mM dNTP, 2 mM NAD, 1.1 M betaine, 1× Ampligase buffer, 5 U Ampligase (Epicentre, Madison, WI, USA) and 0.8 U Phusion polymerase (NEB, Ipswich, MA, USA) was added to the reaction and incubated at 56°C for 60 minutes followed by 68°C for 20 minutes. To completely eliminate linear DNA molecules, 2 μl of a mixture of total of six exonucleases including 3.5 U exo I (Affymetrix, Santa Clara, CA, USA), 18 U exo III (Affymetrix), 4U exo T7 (Affymetrix), 0.4 U exo T (NEB), 3 U RecJf (NEB) and 0.2 U lambda exo (Epicentre, Charlotte, NC, USA) was added to the reaction and incubated at 37°C for 30 minutes, 80°C for 10 minutes and 95°C for 5 minutes. Total time for multiplex target capture is approximately 4 hours (Figure 1).

### Sequence library construction and sequencing

The circularized DNA molecules with captured targets were multiplex-amplified using custom-designed universal primer pairs directed at the probes' common backbone, which also included Illumina (San Diego, CA, USA) sequencing adapters attached at the ends of these primers. Two separate PCRs were performed with primers (P5-GAT CTT AAC CCT CAC TAA AGG GAG GC and P7-index-GAT CCC AAT TTA GGT GAC ACT ATA GGC GG; and P5-GAT CCC AAT TTA GGT GAC ACT ATA GGC GG and P7-index-GAT CTT AAC CCT CAC TAA AGG GAG GC) in order to sequence both ends of each amplicon using read 1 and read 2, respectively (Figure 1). For pooling multiple samples per MiSeq run we used a 6 bp index sequence (Illumina). P5 (5'-AAT GAT ACG GCG ACC ACC GAG ATC TAC AC-3') and P7 (5'-CAA GCA GAA GAC GGC ATA CGA GAT-3') are the oligo sequences that hybridize to the MiSeq flow cell. The PCR conditions are as follows: 3 to 5 μl of circularized molecules with captured sequences, 1× Phusion GC buffer (NEB), 0.2 mM dNTP, 0.01 U/μl Phusion polymerase (NEB) and 0.4 μM of each of the two amplification primers listed above in a 50 μl final reaction volume, which was incubated at 98°C for 2 minutes followed by 25 cycles of 98°C for 10 s, 64°C for 20 s, and 72°C for 30 s and a final extension for 5 minutes at 72°C. The PCR products were purified using Mag-Bind EZ Pure (Omega Bio-Tek, Norcross, GA, USA) and concentration was measured using Agilent's bioanalyzer (Agilent Technologies, Santa Clara, CA, USA). Samples were pooled in equal concentration, size selected by Pippin Prep (Sage Science, Inc., Beverly, MA, USA) and sequenced by MiSeq using read 1 and read 2 primers (5'-GAT CTT AAC CCT CAC TAA AGG GAG GCG CGC C-3'/5'-GAT CCC AAT TTA GGT GAC ACT ATA GGC GGC CGC-3') in order to obtain read 1 and read 2 sequences from both ends of inserts. Multiple indexed samples were also pooled in equal amounts for MiSeq sequencing. Total time for library construction is approximately 4 hours. The entire assay time from capture to sequencing start is approximately 8 hours.

### Sequence read processing and single nucleotide variant calling

Raw fastq read sequences were de-multiplexed and blasted (blastall 2.2.17) against the 'insert-only' reference sequence (approximately 1.03 Mb) corresponding to the entire amplicon reference sequence (approximately 1.3 Mb) excluding the landing sites of LPPs. Reads aligned to the insert-only reference sequence with a *P*-value of e-6 were considered unique to the captured target sequences. All reads that had passed this filter were blast against the full amplicon reference sequences and non-match sequences (for example, post-capture amplification sites, sequence adapters) were removed. Only the high scoring segment pair to each amplicon sequence was kept. In the event of multiple high scoring segment pairs, one was chosen randomly. If a read had multiple alignments to the same amplicon, the entire region spanning across multiple alignments was kept. Based on blast alignment information we separated reads into four group of reads: read 1 sense and read 2 antisense (originating from PCR-A templates), and read 1 antisense and read 2 sense (from PCR-B templates). Accordingly, two pairs of fastq files were generated: R1-PCR-A.fastq/R2-PCR-A.fastq and R1-PCR-B.fastq/R2-PCR-B.fastq. In order to retain singleton reads (1.3 to 2.7% of filter-passed reads were unpaired) the first three nucleotides from the original mate of the singleton were kept as a placeholder. The processed fastq files were aligned to the human reference sequence (human_g1k_v37) using the Burrows-Wheeler Aligner (BWA version 0.5.9-r16) using default settings and filtering parameterss for base quality score at 20: bwa aln -n 0.04 -o 1 -e -1 -d 16 -i 5 -l inf -k 2 -M 3 -O 11 -E 4 -q 20 (human_g1k_v37.fasta processed_fastq_file). The resulting PE bam files were realigned using GATK IndelRealigner and single nucleotide variant (SNV) and indel calling was performed with the GATK Unified Genotyper (.vcf output) implemented in our Galaxy pipeline. We computed base level coverage for all target bases from samtools pileup of the bam files produced after indel realignment. Amplicon-level read counts were determined using SAMtools (samtools view -q30 bam exon-region). For large exons that required multiple overlapping LPPs for capture, the overlap region was removed from each amplicon. Mapping quality (MAPQ) filtering was set at 30.

### DNA copy number analysis

We calculated the normalized copy number values for each exon in each sample as follows. First, for each sample run and each of the PE reads (that can come from two different PCRs), we obtained amplicon-level coverage (*A*) as the total count of reads aligned within each amplicon based on the final bam files that were processed as described above. Second, within a sample run and a single PCR, we scale-normalized the amplicon-level coverage *A *using NA12878 as reference. Third, for each amplicon, we took the median of normalized coverage across PCRs and sample repeats when available. We then used the lowess function in *R *to draw smoothed curves for visualizing segments of copy number changes for chromosome 13 for NA03330 and in chromosome × for the OTC family.

## Results and discussion

In the process of optimizing probe construction we discovered effective DNA target capture using double-stranded cLPPs. Initially, this finding was unexpected because cLPPs consist of two fully complementary ssLPPs that co-hybridize and thus would prevent genomic target capture. However, our assay uses an excess of probes over the amount of target (LPP/target ratio of approximately 40:1) and following denaturation at higher temperature (98°C, 3 minutes) and strand separation, probe-target formation may be triggered by chance over the time span of this protocol. An alternative but less likely hypothesis is a strand displacement mechanism, where two strands with partial or full complementarity can hybridize to each other, displacing in the process one or more pre-hybridized strands [30]. In contrast to ssLPP, the process of constructing cLPPs directly from a PCR product (Figure 1a) allows for better quality control and is more time and cost-effective. The major expense in cLPP production is the cost of primers (two 60mers/probe) synthesized using standard column-based synthesis because each probe is produced through individual PCR. However, primer costs are easily amortized, which makes cLPP capture economical at reagent costs of <$1 per gene per sample when studying at least 40 genes in 100 or more samples (Figure S2 in Additional file 1).

The cLPP multiplex capture of thousands of exons in a single tube can be performed within approximately 4 hours. The principal steps of probe hybridization to genomic DNA (2 hours), gap-filling and ligation with probe circularization and target capture (1.5 hours), and linear DNA removal (0.5 hours) are identical to ssLPPs (Figure S1 in Additional file 1). While ssLPPs target only one DNA strand, cLPPs are directed at both the sense and antisense strand (Figure 1b). A post-capture multiplex PCR (1 hour) amplifies the entire capture library followed by purification, size selection (1.5 hours) and direct NGS. There is no need for additional shotgun library construction [18], which is similar to the 'library-free' protocol developed for single-stranded MIPs [13]. A major difference to the MIP assay, however, is that LPPs are capable of increased gap-filling of ≤550 bp, which is at least four times the size of MIP capture products [14,21]. Although there are many benefits to LPPs' large capture sizes (see Conclusions), short-read NGS is still insufficient to generate full-length sequences of LPP libraries. Using the MiSeq instrument (Illumina), we initially produced 151 bp PE reads covering a maximum insert size of 300 bp, but leaving approximately 12% of our amplicons with incomplete coverage (3% of total bases).

To address this and extend read length, we gradually added sequencing cycles beyond the standard 151 bases generated on this instrument. We noted that sequence reads originating from the P5 flow cell adapter (read 1) allowed extended reads of 175 bases (87% of bases >Q30), compared to standard 151 bp reads from the P7 adapter (read 2) (85% of bases >Q30) (Figure S3 in Additional file 1). Similar results were found using the recently improved MiSeq reagent kit for extended PE reads of 250 bp with 87.5% of read 1 bases and 75.7% of read 2 bases at >Q30. To overcome this PE read imbalance in targeted sequencing, we modified the standard PE sequencing protocol of reading the forward and reverse strand of a DNA template during one PE read. Our new library preparation protocol utilizes two separate multiplex PCRs (instead of the previous single PCR) during which the sequence adaptors swap positions at the ends of templates (Figure 1c). After pooling the two PCRs, this allows both forward and reverse sequencing of both the sense and antisense strand of a DNA template in a single PE sequencing run. The four unique sequence reads per template are accurately traceable during sequence alignment and can be used separately or in combination for analysis. This approach, termed reciprocal paired-end (rPE) sequencing, enabled covering larger inserts (for example, 350 bp with 175/150 rPE sequencing) and reduced the number of incompletely covered amplicons to 5% (1% of total bases).

**Figure 2:**
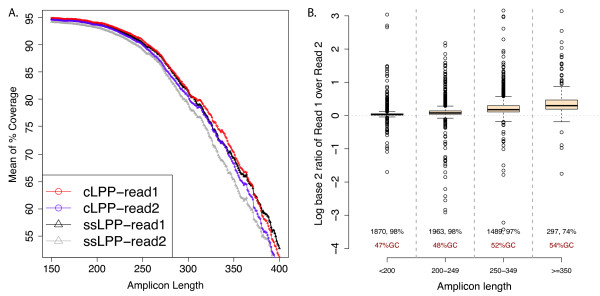
**Coverage distribution across target regions**. **(a) **Cumulative mean percent base coverage across 5,619 targets captured using cLPPs and ssLPPs, respectively, and shown separately for sequence read 1 and read 2. All bases have a minimum of 10× coverage. **(b) **Log ratio of coverage of read 1 and 2. Each boxplot corresponds to coverage distribution of a group of amplicons within a defined size range with number of amplicons, percent bases covered (≥10×) and average GC content shown for each group. All groups present a statistically significant distribution different from each other and each maintains a mean significantly different from 0.

We next compared the ability of cLPPs and ssLPPs for capturing 5,471 exons (524 genes, 1.3 Mb) [18]. NGS library preparation was performed using our new protocol for rPE library amplification followed by MiSeq sequencing. To ensure comparability, we constructed cLPPs and ssLPPs from the same PCR products and performed all experiments under identical conditions. The two barcoded libraries (cLPPs #4 and ssLPPs #3, Additional file 2) were sequenced in one MiSeq run that generated a similar number of 250 PE reads for each library. We used HapMap sample NA18507 with a known genotype [31] to compare the two capture methods. Based on several parameters by which assay performance can be measured [2], cLPPs performed equally or significantly better in every test (Additional file 2), including target specificity (*P *< 0.05) and coverage uniformity (Figure S4 in Additional file 1). At similar amplicon and base coverage, we observed significantly improved mean performance (*P *< 0.05) for the detection of both heterozygous and homozygous sample SNVs for cLPPs compared to ssLPPs. In addition, we analyzed separately the base coverage of read 1 and 2 in relation to target length (Additional file 3). For both capture probes, base coverage gradually declined starting at approximately 150 bp (Figure 2a), which was more pronounced for read 2 and most evident for larger amplicons (Figure 2b). For amplicons >350 bp, rPE sequencing increased base coverage by 2.7% compared to standard PE sequencing (Figure S5 in Additional file 1). Most recently, we tested different DNA polymerases (for example, Phusion, KAPA) during rPE library preparation (cLPPs #5 to 7, Additional file 2), further improving assay performance. A summary of the performance parameters for cLPP capture is shown in Table 1.

**Table 1:** Performance of cLPP target capture and sequencing

(i) Sensitivity; percentage of the target bases that are represented by one or more reads	98.7% of all exons and 97.8% of all target bases at >20× coverage (0.012× mean coverage)
(ii) Specificity; percentage of sequence reads that map to the intended targets (5,471 exons)	98.1% mapped target reads confirming the high on-target specificity of LPP capture
(iii) Accuracy; base calling concordance to known sample SNVs	>99% concordance rate for both heterozygous and homozygous SNVs with coverage of 97.9%; sample SNVs at >20× coverage (0.012× mean)
(iv) Uniformity; variability in sequence coverage across target regions	91% of capture products were distributed within a 50-fold range (94% within 100-fold)
(v) Reproducibility; or how closely results obtained from independent samples correlate	r = 0.93 rank-order correlation between two different HapMap samples (Figure S8 in Additional file 1); 1.15% SD (*P *= 0.01) for detecting heterozygous SNV (>20×) in five cLPP capture experiments.
(vi) Cost of LPP target capture	$86 per sample for 100 genes in 100 samples or $16.70 per sample for 100 genes in 1,000 samples (Figure S2 in Additional file 1).
(vii) Ease of use and time effort	<8 hours target capture and library preparation ('sequencing-ready'), <24 hours MiSeq and approximately 6 hours variant calling (524 genes). Total time: 38 hours
(viii) DNA amount required per experiment	>50 ng of genomic DNA
(ix) Multiplexing of candidate genomic targets and of DNA samples	Multiplex target capture of 524 genes per sample; sample multiplexing of 7 capture libraries per MiSeq run (#B, Additional file 2).

Performance parameters are adapted from [2] and calculated for cLPPs based on: (i to iv) multiplex targeted sequencing of 524 genes using cLPPs and MiSeq for NA18507 (experiment #7, Additional file 2); (v) comparison of two independent sample preparations (NA18507 and NA12878, experiment #6) and by estimating the standard deviation (SD) across five cLPP capture experiments (experiment #2, 4 to 7, Additional file 2).

In order to compare cLPP capture to other methods, we used data from a recent performance comparison of three commercial WES platforms [23]. For specificity, this study found that 9.3% of Nimblegen, 12.8% of Agilent and 35.6% of Illumina reads uniquely mapped to off-target regions (see Figure 3a in [23]). In comparison, only 1.9% of our reads mapped off-target (Table 1). For accuracy, our method had a 99.4% concordance rate for known sample SNVs, which was 99.3% for Agilent, 99.5% for Nimblegen and 99.2% for Illumina. Thus, at similarly high accuracy, LPP capture has a significantly higher target specificity (98%) than WES and requires less sequencing to generate adequate target coverage, which makes sequencing more economical [2]. Another method that is perhaps better comparable to LPPs was developed by RainDance Technologies and utilizes an enrichment approach based on microdroplet multiplex PCR [8]. Using this technology, 79 to 84% of uniquely mapping reads aligned to targeted amplicons (specificity) and 90 to 97% of targeted bases were covered within a 25-fold abundance range (uniformity) [8]. Although RainDance achieved a better uniformity, LPP capture has a higher target specificity, lower DNA sample input requirements [32] and does not require expensive laboratory equipment other than a PCR instrument. The high data quality, low assay costs and the ability for multiplexing both targets and samples make cLPPs and the related MIP technology particularly suitable for disease-targeted sequencing of panels of genes (for example, tens to thousands of genes) in large numbers of people.

We also sought to identify copy-number variation at targeted genes directly from sequencing data. Copy number variant detection in targeted NGS is challenging due to the small size and non-contiguous nature of target regions, and the technical variability in coverage that can confound it [33-35]. As described by Li *et al. *[35], we calculated standard deviations of log ratios of coverage for a test and reference sample for bins (200 exons) of regions with similar coverage. Compared to WES [35], cLPP capture data presented a four-fold increase in overall base coverage with a significantly lower standard deviation (SD) of 0.3 to 0.15 for exons at high coverage of 2^8 ^to 2^12 ^(SD 0.35 at 2^8 ^for WES), and lower coverage of 2^3 ^with SD of 0.5 (SD of 0.8 for WES) (Figure S6 in Additional file 1). Using our panel of 524 genes distributed across the genome, we correctly detected a chromosomal aneuploidy (trisomy 13), and confirmed the deletion of nine of ten exons of the ornithine transcarbamylase (*OTC*) gene (Xp11.4) in a boy with OTC deficiency [18], while his mother had a single copy of the same nine exons (Figure S7 in Additional file 1). These results confirm the robust and reproducible performance of LPPs and demonstrate their ability to accurately preserve a sample's genome information in order to detect both chromosomal and focal genomic rearrangements (for example, intragenic deletion) at high resolution from targeted NGS data.

## Conclusions

Our data demonstrate multiplex target capture with double-stranded, complementary LPPs performed better than single-stranded LPPs and can be built in less than half the time of traditional probe construction. Next efforts to improve capture are directed at the critical steps of probe-target hybridization and gap-filling efficiency. Hybridization kinetics can be optimized using new design algorithms and by replacing failed probes [14]. In comparison to MIPs, the ability of LPPs for increased gap-filling (≤550 bp) provides additional flexibility for shifting the probes' capture arms flanking a target. This can help to: (i) maximize capture length to take advantage of increasingly longer NGS reads; (ii) cover most human exons using a single probe, making sequencing more economical; (iii) avoid common SNVs in the probes' annealing regions that can lead to amplification failures or overlooked sample variants; and (iv) reduce the relative GC content of hard-to-capture targets. Notably, 44% of the exons that escaped detection compared to only 22% of the captured exons had a GC content larger than 65%. While both strands in a linear duplex DNA have identical GC content, stable probe-target hybrids may form at only one strand. This provides increased likelihood for target capture using cLPPs compared to single-stranded probes targeting only one strand. The formation of a four-stranded hybrid complex [36] of cLPPs with a target DNA is rather unlikely due to the slowness in rehybridization kinetics. To utilize the continuously improving NGS read length, LPPs were designed to capture increasingly larger regions [17]. However, the PE read imbalance detected here using MiSeq is a particular problem for targeted sequencing as only two unique reads are generated per template. To address this, we developed rPE library amplification, which doubled the number of unique sequence reads per template from two to four in a single PE read. Using two separate PCRs minimizes random errors from PCR amplification [37], and extended rPE reads can help to distinguish true variants from sequence errors by confirming their occurrence on both strands [38]. The rPE strategy is applicable to library preparation protocols of other targeted sequencing methods.

## Abbreviations

bp: base pair; cLPP: complementary long padlock probe; LPP: long padlock probe; MIP: molecular inversion probe; NGS: next-generation sequencing; OTC: ornithine transcarbamylase; PE: paired-end; rPE: reciprocal paired-end; SNV: single nucleotide variant; ssLPP: single-stranded long padlock probe; SD: standard deviation; WES: whole-exome-sequencing.

## Competing interests

The authors declare that they have no competing interests.

## Authors' contributions

PS, WW, RWD and CS designed the research, PS performed the research and sequenced the libraries, PS, WW, AC, YF and CS analyzed data, and PS, WW and CS wrote the paper, which all authors edited. All authors read and approved the final manuscript.

## Supplementary Material

Additional file 1 **Figures S1 to S8**. Figure S1: comparison of ssLPP and cLPP construction. Figure S2: an estimate for reagent cost for cLPP capture. Figure S3: the sequence read qualities for 175/150 PE sequencing. Figure S4: coverage uniformity for ssLPP and cLPP capture. Figure S5: coverage difference for standard and reciprocal PE sequencing. Figure S6: log-ratio variations versus log coverage in targeted NGS data. Figure S7: detection of chromosomal and focal CNV. Figure S8: reproducibility in cLPP capture. Click here for file

Additional file 2 **Table giving the performance of different LPP captures and sequencing**. Click here for file

Additional file 3 **Coverage of read 1 and 2 derived from rPE library amplification**. Click here for file
