# Supplementary material for: Multiplex target capture with double-stranded DNA probes
Source: Genome Med. 2013 May 29;5(5):50. doi: 10.1186/gm454 (PMC3706973; doi:10.1186/gm454)
Supplement: Additional file 1 — Figures S1 to S8. Figure S1: comparison of ssLPP and cLPP construction. Figure S2: an estimate for reagent cost for cLPP capture. Figure S3: the sequence read qualities for 175/150 PE sequencing. Figure S4: coverage uniformity for ssLPP and cLPP capture. Figure S5: coverage difference for standard and reciprocal PE sequencing. Figure S6: log-ratio variations versus log coverage in targeted NGS data. Figure S7: detection of chromosomal and focal CNV. Figure S8: reproducibility in cLPP capture. [file gm454-S1.PDF]

Additional File 1

Supplementary Figure 1: Construction of ssLPPs and cLPP. The protocol of producing single-stranded LPPs involved six principle steps[1,2], while double-stranded LPPs can be constructed in two steps directly from the double-stranded probe precursor by enzymatic end trimming and 5' phosphorylation. cLPP construction is greatly simplified and easy to control. Time effort for constructing ssLPP is ~8 hrs and ~4 hrs for cLPP.

Supplementary Figure 2: Reagent cost estimate for cLPP target capture. The expenses are the total reagent cost per sample for cLPP construction including oligonucleotide primers ($7.70 per probe), target capture reagents ($2), library preparation including multiplex PCR ($2), PCR quantification using Agilent Bioanalyzer ($2) and size selection using Sage Science Pippin Prep ($3) assuming four barcoded sample libraries per MiSeq run. The cost estimate does not include technician labor cost, machine purchase and operating costs, computation and analysis costs. The total cost for target capture and library preparation ("sequencing-ready") are increasingly economical with <$1 per gene and sample when studying 10 or more genes in at least 1,000 samples, or 40 or more genes in at least 100 samples.

Supplementary Figure 3: Read 1 and read 2 base qualities (Q scores) for 175/150 PE sequencing. Quality score (Q) across all bases in all reads (y axis) at base position in reads (x-axis). There is an inverse relationship of Q score and read length with a gradual decline in base quality towards the end of reads. This decline is more prominent for read 2 (right) than 1 (left).

Supplementary Figure 4: Coverage uniformity for cLPP and ssLPP capture. Distribution of log base 2 coverage for 5,619 amplicons captured using cLPPs (upper panel) and ssLPPs (lower panel). Each bar represents a 2-fold difference in coverage with the values of the two most frequent coverage ranges for each capture method shown in the bars. The dashed line represents the coverage density. Overall, 91% of cLPP capture products distributed within a 50-fold range (94% within 100-fold) compared to 89% within 50-fold for ssLPPs (92% within 100-fold).

Supplementary Figure 5: Mean coverage difference for standard and reciprocal PE sequencing. The percent base coverage for groups of amplicons within a defined size range at 0.1x mean coverage for traditional PE sequencing (PCR 1 in blue, PCR 2 in red) and reciprocal PE sequencing (in green). For large amplicons >350bp, rPE sequencing increased the base coverage by 2.7% compared to standard PE sequencing.

Supplementary Figure 6: Log-ratio variations versus log coverage. The log ratio is based on the coverage of one sample (NA18507) versus that of a reference sample (NA12878). Log ratios are calculated following the procedure described by Li *et al.*[3]. Each dot corresponds to a group of 200 exons with similar coverage for which the corresponding mean log base coverage (amplicon-level coverage divided by a mean amplicon length of 230) and the standard deviation of log ratios is calculated. A total of 4689 exons were included in the analysis. At 2^8 exon coverage, the standard deviation of LPP capture is comparable to exome capture results (0.3 and 0.35, respectively). Standard deviation of LPP capture decreased to 0.15 at 2^{12} exon coverage. Li *et al.*[3] did not present results from exome capture >2^8 exon coverage.

Supplementary Figure 7: Detecting CNVs from targeted sequencing data. (**a**) NA03330 carries an extra copy of chromosome 13 (karyotype 47XY,+13) and NA18507 is a healthy control (46XY). Circles represent 5,619 exons captured across 23 chromosomes including 166 exons on chromosome 13, vertical lines indicate chromosome boundaries, and the two solid horizontal lines are smoothed curves (windows of 40 exons) of normalized copy numbers at exon level. (**b**) A male child with OTC deficiency (OTC son) and his healthy mother (OTC mom) share a single-copy

deletion of 9 of 10 OTC exons (Xp21.1). Normalized copy numbers are shown for a 12.9 Mb genomic interval containing all 77 exons of five genes (GK, CYBB, OTC, MAOA, MAOB).

Supplementary Figure 8: Concordance of cLPP capture experiments. Shown is a correlation of read depth per amplicon across 5,316 amplicons (autosomes only) in 2 samples (NA18507 and NA12878), which were captured using cLPPs, prepared in parallel, pooled and sequenced in the same MiSeq run. The high concordance (r=0.927) shows the reproducibility of the capture efficiency between independent sample preparations.

References

1. Krishnakumar, S. *et al.* A comprehensive assay for targeted multiplex amplification of human DNA sequences. *Proc Natl Acad Sci U S A* **105**, 9296-301 (2008).
2. Shen, P. *et al.* High-quality DNA sequence capture of 524 disease candidate genes. *Proc Natl Acad Sci U S A* **108**, 6549-54 (2011).
3. Li, J. *et al.* CONTRA: copy number analysis for targeted resequencing. *Bioinformatics* **28**, 1307-13 (2012).

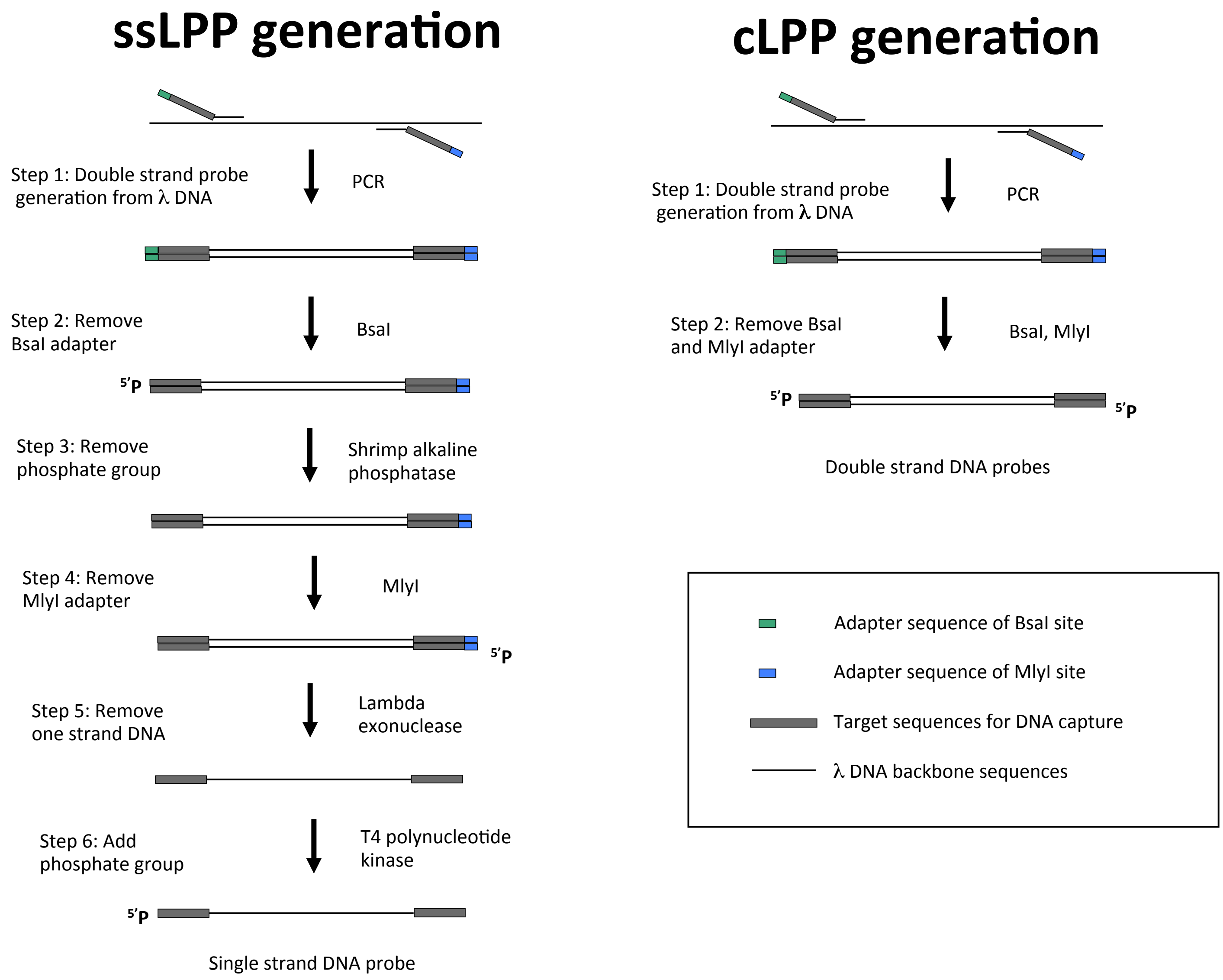

Supplementary Figure 1: Construction of ssLPPs and cLPP.

Supplementary Figure 2: Reagent cost estimate for cLPP target capture

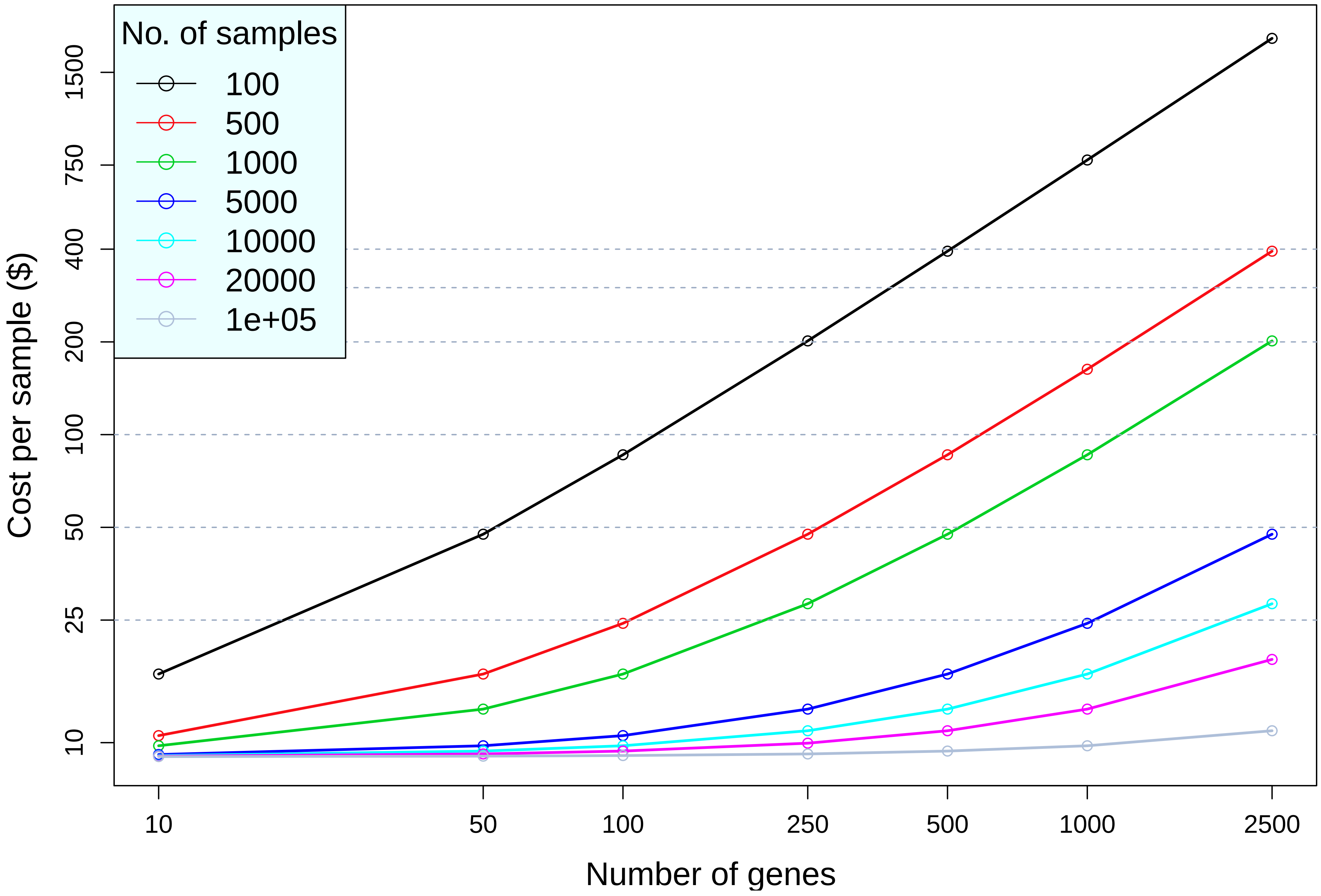

Read 1 (175bp)

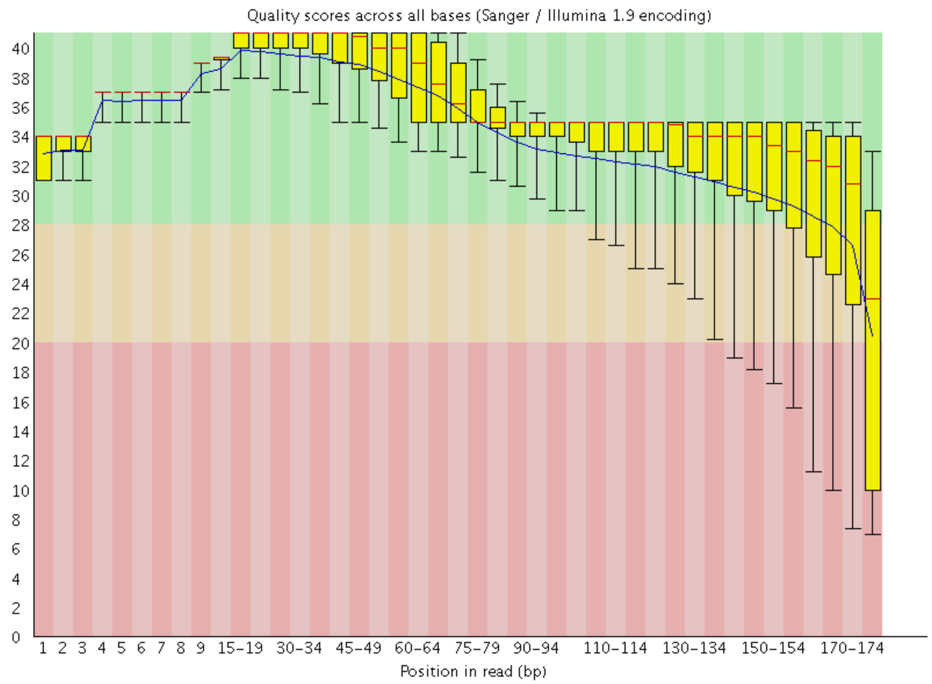

Q >= 30: 87.2%

Read 2 (151bp)

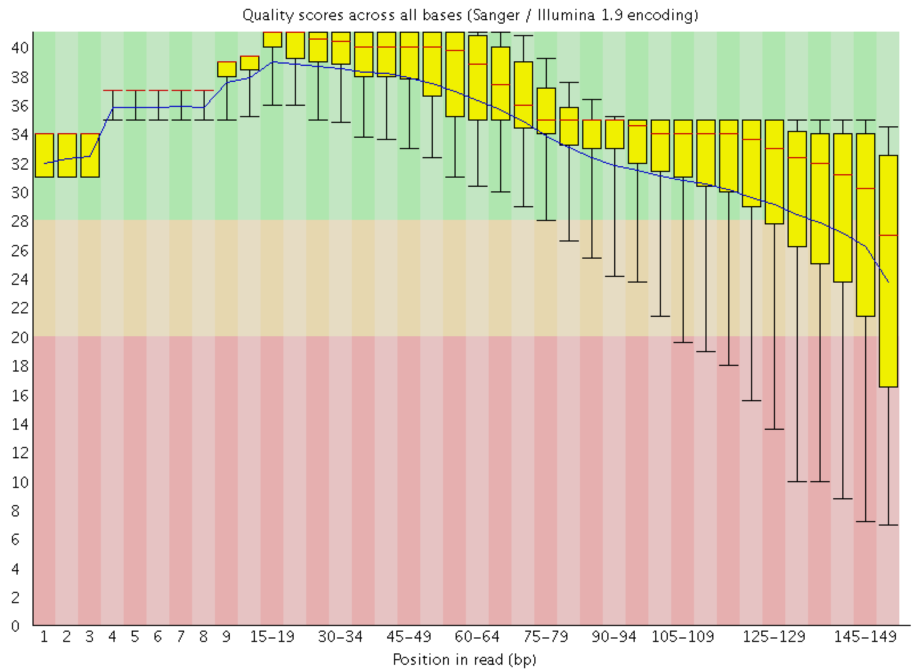

Q >= 30: 85.1%

Cluster: 770K/mm^2
Total Reads: 2x6.42M
% pass filter (PF): 94.5%

Supplementary Figure 3: Read 1 and read 2 base qualities (Q score) for 175/150 PE sequencing.

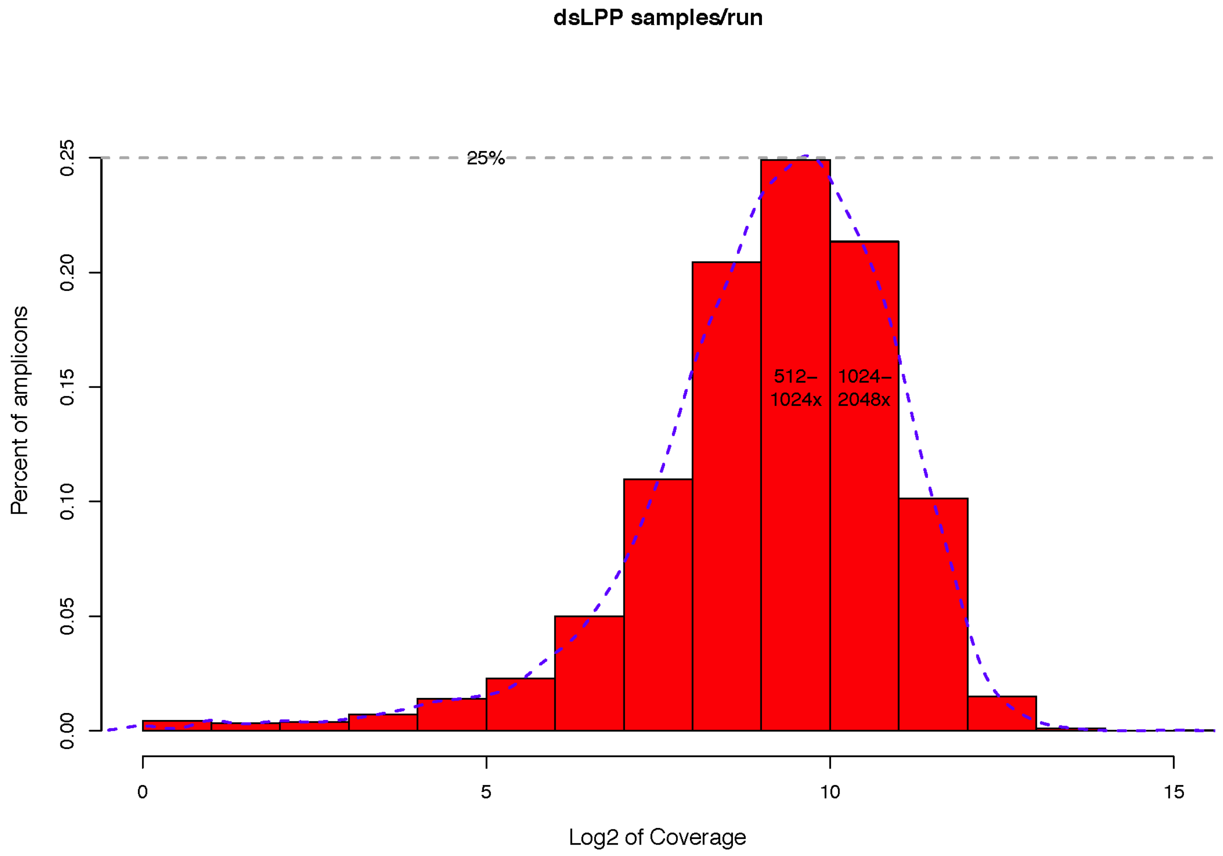

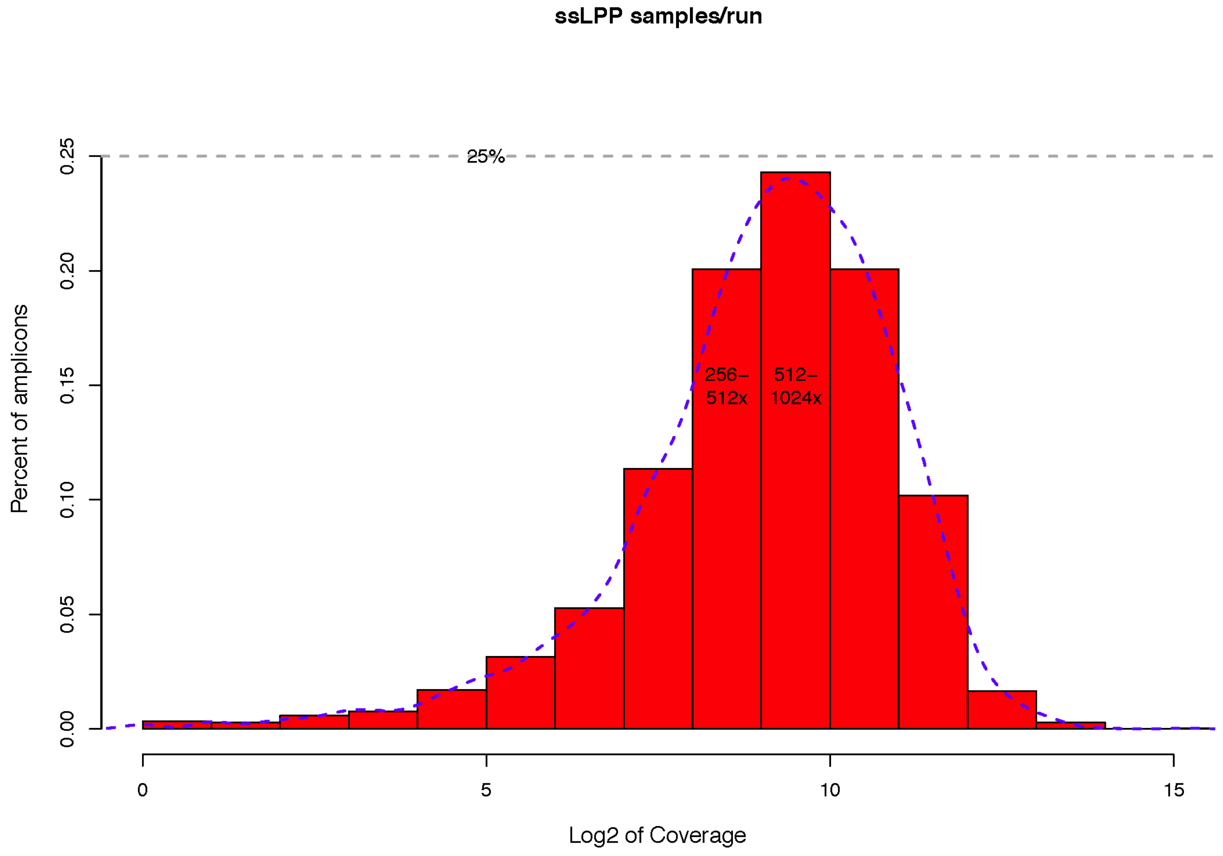

Supplementary Figure 4. Coverage uniformity for cLPP and ssLPPcapture.

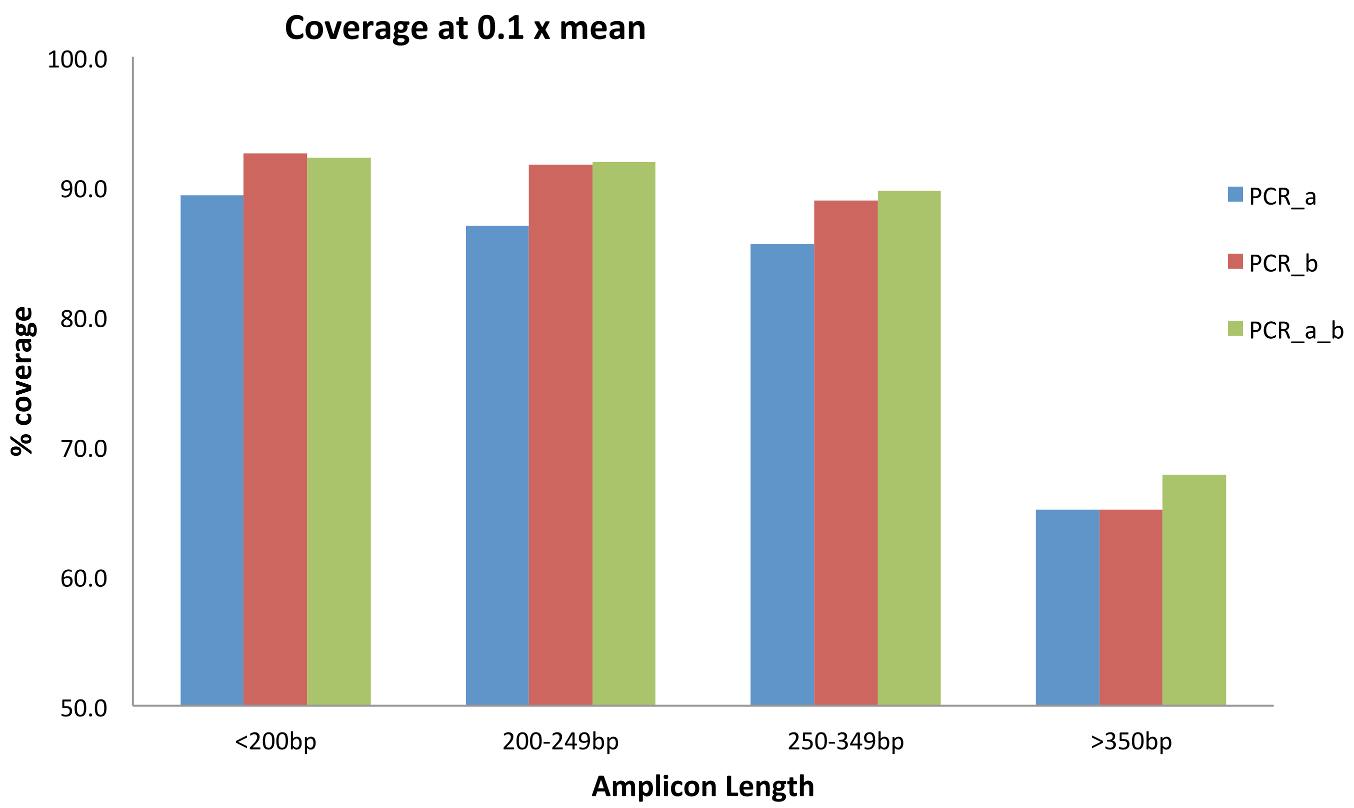

0.1x Mean	<200bp	200-249bp	250-349bp	>350bp
PCR_a	89.3	87.0	85.6	65.1
PCR_b	92.6	91.7	88.9	65.1
PCR_a_b	92.2	91.9	89.7	67.8

Supplementary Figure 5: Mean coverage difference for standard and reciprocal PE sequencing.

Supplementary Figure 6: Log-ratio variations versus log coverage.

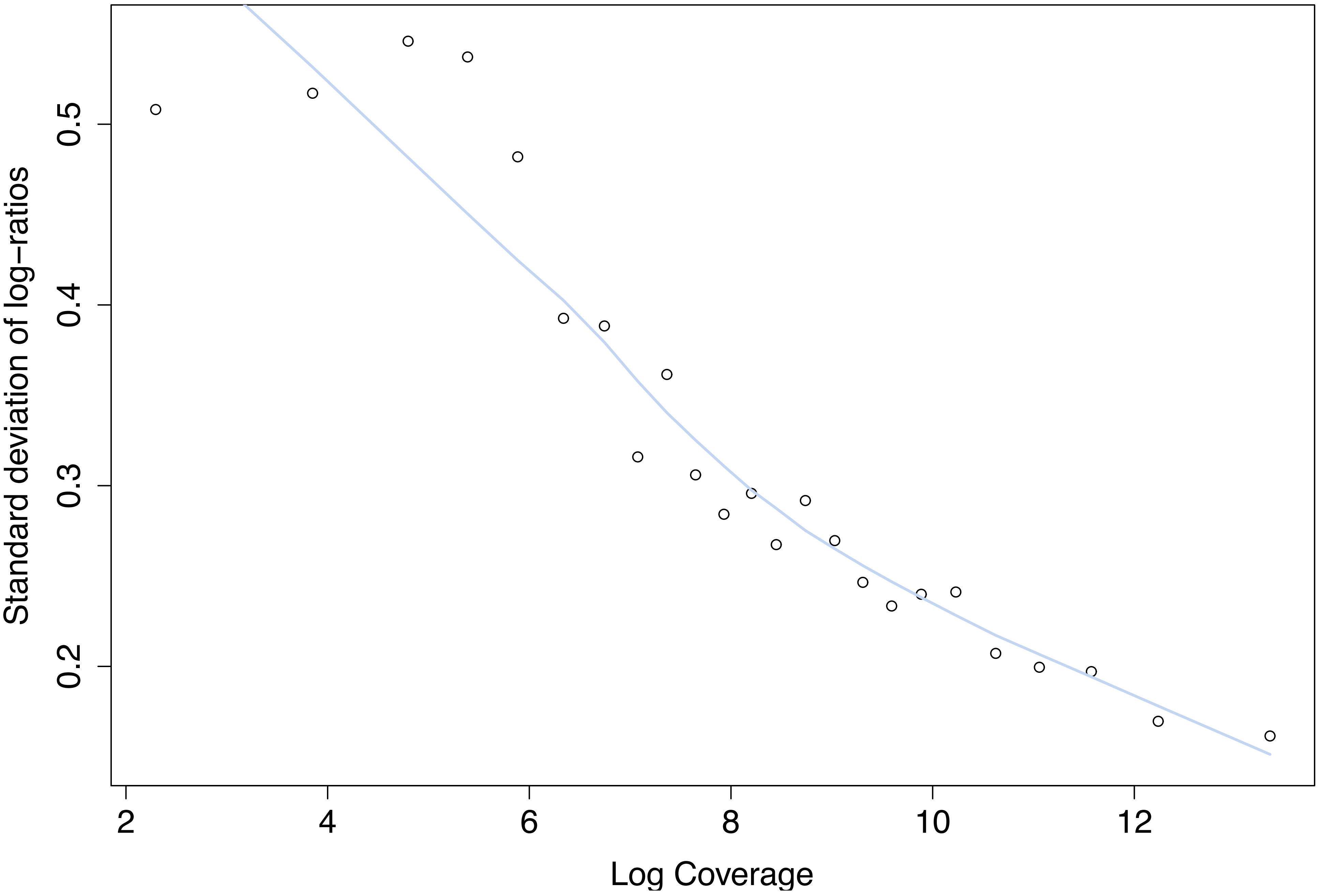

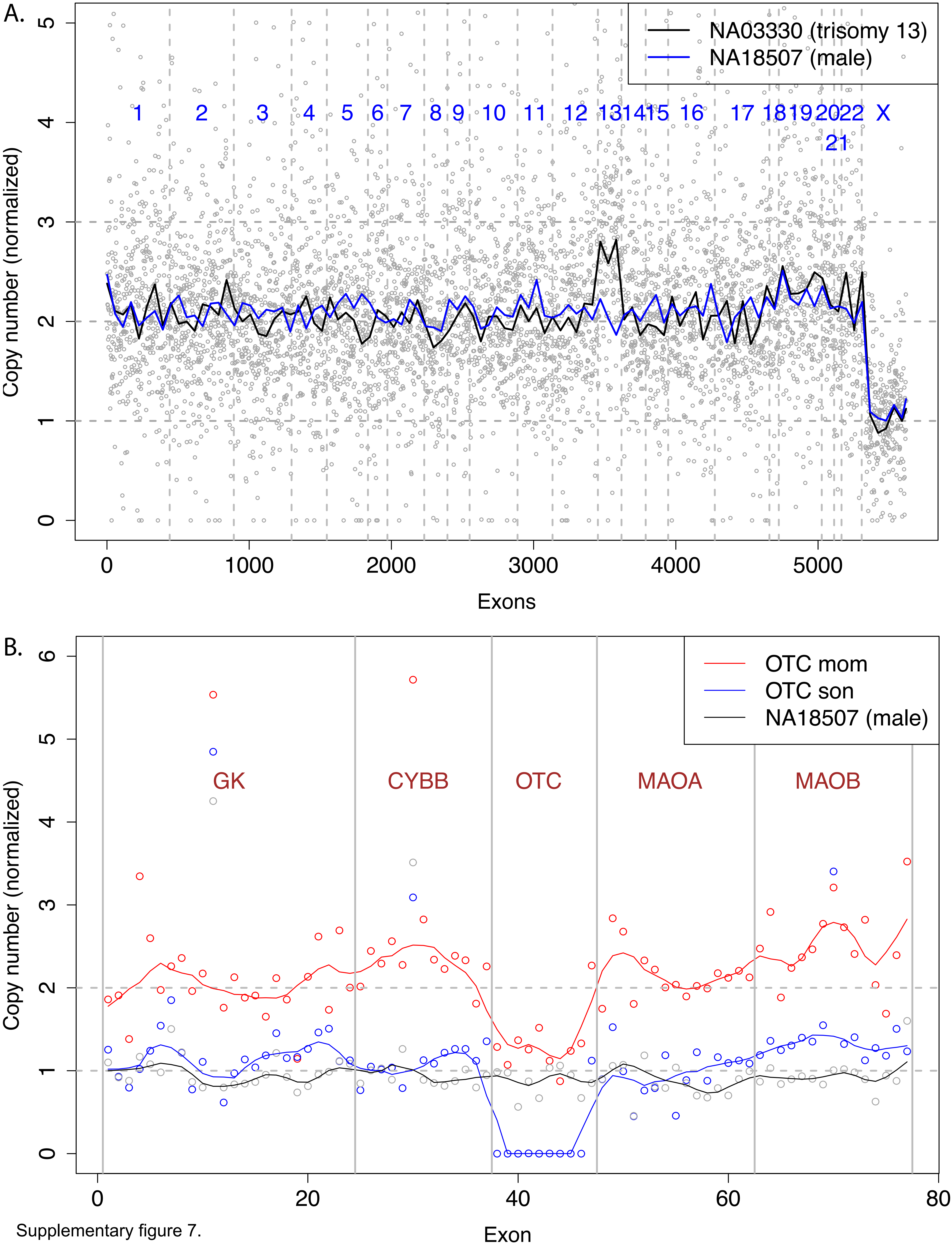

Supplementary figure 7.

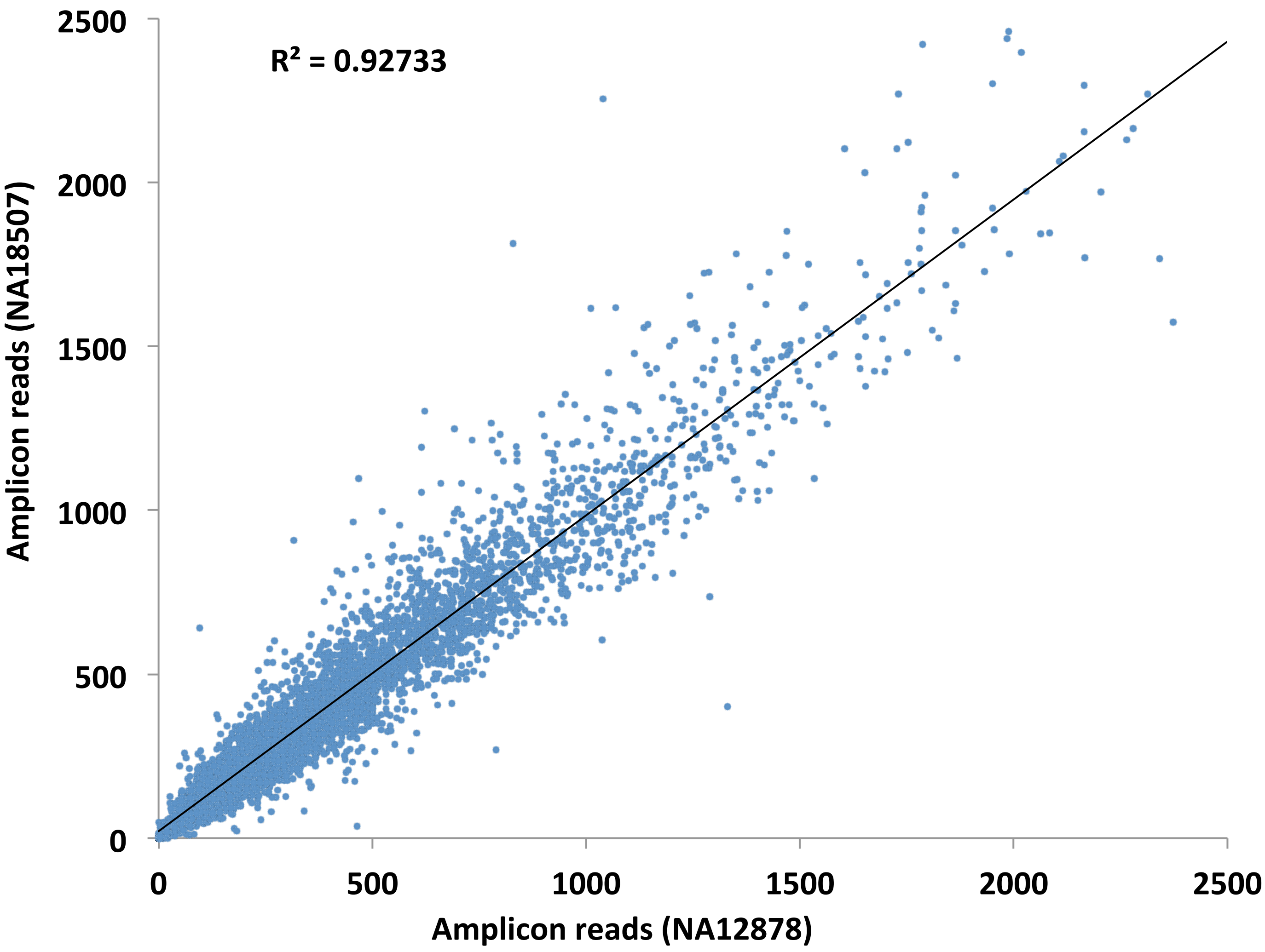

Supplementary Figure 8: Reproducibility of two samples (NA12878 and NA18507)
